# LC-MS/MS-Based Metabolomics and Multivariate Statistical Analysis Reveal the Mechanism of *Rhodotorula mucilaginosa* Proteases on Myofibrillar Protein Degradation and the Evolution of Taste Compounds

**DOI:** 10.3390/foods14111867

**Published:** 2025-05-24

**Authors:** Tianmeng Zhang, Qiang Xia, Daodong Pan, Yangying Sun, Ying Wang, Jinxuan Cao, Ren-You Gan, Changyu Zhou

**Affiliations:** 1 Zhejiang Key Laboratory of Intelligent Food Logistic and Processing, Zhejiang-Malaysia Joint Research Laboratory for Agricultural Product Processing and Nutrition, College of Food Science and Engineering, Ningbo University, Ningbo 315211, China; 2211390087@nbu.edu.cn (T.Z.); xiaqiang@nbu.edu.cn (Q.X.); daodongpan@163.com (D.P.); sunyangying@nbu.edu.cn (Y.S.); 2 Key Laboratory of Geriatric Nutrition and Health, Beijing Technology and Business University, Ministry of Education, Beijing 100048, China; wang-ying@btbu.edu.cn (Y.W.); caojinxuan@btbu.edu.cn (J.C.); 3 Department of Food Science and Nutrition, The Hong Kong Polytechnic University, Hong Kong 999077, China; 4 Research Institute for Future Food, The Hong Kong Polytechnic University, Hong Kong 999077, China

**Keywords:** myofibrillar proteins, *Rhodotorula mucilaginosa*, proteases, γ-glutamyl peptides, taste attributes

## Abstract

*Rhodotorula mucilaginosa* plays a key role in developing the taste of dry-cured ham, while the mechanism of *Rhodotorula mucilaginosa* proteases on myofibrillar protein (MP) hydrolysis and the evolution of taste substances has not been studied. The enzymatic characteristics, hydrolysis capacities for MPs, free amino acid contents, metabolite compositions, and taste attributes were investigated during the interactions of MPs and proteases. The proteases of *R. mucilaginosa EIODSF019* (RE) and *R. mucilaginosa XZY63-3* (RX) showed high hydrolytic activities at the conditions of pH 5.0~7.0 and 30~40 °C. Compared with RX, RE showed a lower Michaelis constant (Km) value and a better affinity for protein substrates. RE showed a higher capability to degrade myosin and actin compared with RX and *P. kudriavzevii XS-5* proteases (PK). The microtopography of enzyme-treated MPs in RE presented a smoother surface and lower root mean square roughness than that in RX and PK. The total content of free amino acids significantly increased from 0.34 mg/100 mL of CK to 17.10 mg/100 mL of RE after 4 h of hydrolysis of MPs. Sixty-two metabolites were identified by LC-MS/MS, and γ-glutamyl peptides were the main components of MP hydrolysates. Sensory scores of umami, richness, and aftertaste showed the largest values in RE among these groups. Partial least squares discriminant analysis and correlation network demonstrated that γ-Glu-Lys, γ-Glu-Tyr, γ-Glu-Glu, γ-Glu-His, γ-Glu-Leu, γ-Glu-Cys, γ-Glu-Ala, and γ-Glu-Gln were positively correlated with the improvements of umami, richness, and aftertaste in RE.

## 1. Introduction

Taste is considered the most essential quality attribute of dry-cured ham, appreciated by consumers because of the distinctive organoleptic characteristics [1,2]. During the fermentation–ripening of dry-cured ham, protein degradation is a primary process in the development and formation of taste [3,4]. Myofibrillar proteins (MPs), which account for approximately 50% of meat proteins, are the primary component. Moreover, MP degradation significantly contributes to the development of flavor in the final product [5]. The majority of researchers believe that protein hydrolysis is primarily attributed to endogenous and microbial proteases during dry-cured ham production. Several studies have highlighted the activities of endogenous proteases on the degradation of MPs during the fermentation–ripening process of ham [6,7,8]. However, during the later stage of fermentation, the activities of certain endogenous enzymes may be inhibited due to high salt, low humidity, and extreme pH conditions. This inhibition reduces the efficiency of endogenous enzymes in hydrolyzing proteins and prevents the taste formation of the final products [9]. Exogenous proteases sourced from indigenous microorganisms can compensate for these deficiencies of endogenous proteases, and they play a pivotal role in hydrolyzing proteins during the fermentation of ham [10]. However, limited research focuses on the role of indigenous microorganisms in the hydrolysis of structural proteins and the evolution of taste compounds in dry-cured ham.

Yeast is a significant component of the fungal flora present during the dry-ripening of ham [11]. It is present throughout the entire process and plays a crucial role in developing the flavor profile of dry-cured ham. This contribution is largely attributed to its enzyme system, which comprises a considerable quantity of proteolytic enzymes that markedly facilitate the accumulation of taste substances in dry-cured ham [12,13]. Some studies have shown that *Debaryomyces hansenii*, *Saccharomyces cerevisiae*, and *Candida zeylanoides* can serve as starter cultures during the fermentation of meat products to significantly promote the formation of characteristic volatile compounds by hydrolyzing muscle proteins [12,14,15,16]. Although the relationship between yeast community succession and the evolution of characteristic volatile compounds has been demonstrated, there is still a lack of a comprehensive understanding of the enzymatic characteristics of yeast extracellular proteases as well as the mechanism of extracellular protease-mediated hydrolysis of muscle proteins.

*Debaryomyces hansenii*, *Rhodotorula mucilaginosa*, *Yarrowia lipolytica*, and *Saccharomyces cerevisiae* are commonly found in dry-cured hams [13]. *Rhodotorula mucilaginosa* is a highly exploitable yeast and has attracted a great deal of research interest [17,18]. Current research has demonstrated that *Rhodotorula mucilaginosa* has an excellent capacity to produce endo-1,4-glucanases and lipase, which are widely used in agriculture and the chemical industry [18]. However, little research has been conducted to investigate the effect of *Rhodotorula mucilaginosa* on the proteolysis during the fermentation of dry-cured ham, particularly the mechanism of *R. mucilaginosa* on the hydrolysis of meat proteins and the evolution of taste compounds is still unclear. Thus, it is necessary to investigate the properties of *R. mucilaginosa* proteases and to further study the capacity to degrade MPs and develop the evolution of taste compounds.

Therefore, the objective of this study was to characterize the enzymatic properties and the proteolytic ability of the proteases of *Rhodotorula mucilaginosa EIODSF019* (RE) and *Rhodotorula mucilaginosa XZY63-3* (RX) isolated from dry-cured hams. Furthermore, the evolution of taste compounds during the hydrolysis process of MPs induced by *Rhodotorula mucilaginosa* proteases was investigated. The metabolites of MP hydrolysates were identified by LC-MS/MS, and the key metabolites were evaluated using partial least squares discriminant analysis (PLS-DA). The relationship between key metabolites and palatability attributes was also explored using the electronic tongue and correlation analysis.

## 2. Materials and Methods

### 2.1. Isolation and Identification of Yeast Strains

Jinhua ham samples (10 g) were minced and homogenized with 90 mL of sterile saline (0.9%, *w*/*v*). Gradient dilutions were plated on yeast extract peptone dextrose medium/YPD medium (with 2% skimmed milk powder and 0.01% chloramphenicol) and incubated at 28 °C for 3 d. Strains with visible hydrolysis circles were purified and identified. These strains were inoculated into YPD liquid medium and incubated for 3 d at 140 rpm. The DNA was extracted, and polymerase chain reaction (PCR) amplification was carried out using NL1 and NL4 primers (5′-GCATATCAATAAGCGGAGGAAAAG-3′ and 5′-GGTCCGTGTTTCAAGACGG-3′, respectively). The PCR products were subjected to separation via 2% agarose gel electrophoresis. Subsequently, they were sequenced by Sangon Biotech and then compared with sequences in the NCBI database for homology analysis. A phylogenetic tree was constructed in MEGA 11.0 to determine taxonomic status (Figure S1). Selected yeasts with large hydrolysis circles were identified as *Rhodotorula mucilaginosa EIODSF019*, *Rhodotorula mucilaginosa* XZY63-3, *Debaryomyces hansenii JNC-14*, and *Yamadazyma mexicana YF17145* (>99% similarity).

### 2.2. Measurement of Protease Activities

Yeast proteases were prepared as described by Li et al. [5]. *Rhodotorula mucilaginosa EIODSF019*, *Rhodotorula mucilaginosa* XZY63-3, *Debaryomyces hansenii JNC-14*, *Yamadazyma mexicana YF17145*, and commercial yeast (*Pichia kudriavzevii XS-5*) were incubated in liquid culture for 3 d at 28 °C. Supernatants were collected, and proteases were precipitated with 80% ammonium sulfate. The proteases were solubilized in 0.02 M phosphate-buffered saline (PBS, pH 7.0) and subsequently adjusted to a concentration of 10 mg/mL using the BCA method, and then stored at 4 °C for further analysis.

Protease activities were measured following the method described by Li et al. [5], with some modifications. Briefly, 1 mL of protease solution was combined with 1 mL of 2% (*w*/*v*) casein substrate and incubated at 40 °C for 10 min, then terminated with 2 mL of 0.4 M TCA. The resulting mixture was then centrifuged at 8000× *g* to collect the supernatant. Subsequently, the supernatant (1 mL) was added to a solution containing 5 mL of Na_2_CO_3_ and 1 mL of 1 M Folin-Phenol Reagent (Beyotime, Shanghai, China), followed by thermal incubation at 40 °C for 20 min. Absorbance measurements were performed at 680 nm using a microplate reader (Thermo Scientific™ Multiskan SkyHigh, Waltham, MA, USA). The control group was the same as that of the experimental group except that trichloroacetic acid (TCA) was added prior to inactivating the protease solution, and the protease activities were calculated from the tyrosine standard curve (0–50 μg/mL). The production of 1 μg of tyrosine per minute of hydrolysis of casein was defined as a unit of enzyme activity, which was expressed as U/mL or U/g.

### 2.3. Influence of pH and Temperature on Protease Activities

The buffers (0.1 M), including sodium citrate (pH 3–5), phosphate (pH 6–8), and glycine–NaOH (pH 9–11), were used to evaluate the impact of pH on protease activities. The optimal pH for protease activities was evaluated by using 2% casein as the substrate. The protease solution was thoroughly combined with the substrate at each pH, followed by incubation at 40 °C for 10 min. Similarly, the influences of temperature on protease activities were investigated across the range of 20 to 70 °C. Protease solution and substrate were blended in equal volumes, and then incubated for 10 min at various temperatures. The above results were presented as relative enzyme activities, and the maximum protease activities were 100%.

### 2.4. Impact of Inhibitors and Metal Ions on Protease Activities

The influence of various metal ions and inhibitors on protease activities was examined at pH 7.0 and 40 °C. Briefly, the proteases were solubilized in 1 mmol/L and 10 mmol/L metal ion solutions (Na^+^, K^+^, Ca^2+^, Mg^2+^, Mn^2+^, Cu^2+^, Zn^2+^, and Fe^2+^) or inhibitor (EDTA) to measure the protease activities according to the above method. The protease activities without the addition of any metal ions or inhibitors were expressed as 100%.

### 2.5. Determination of Kinetic Parameters

The determination of enzymatic kinetic parameters was carried out according to the method described by Li et al. [5]. Briefly, the protease solution (10 mg/mL) was reacted with casein substrates of different concentrations (2.5–40 mg/mL) at 40 °C, and the enzyme activities were determined. The Michaelis–Menten equation of the fitted curves was created from the initial measurements, and the maximum reaction rate (Vmax) and the Michaelis constant (Km) of the protease solutions were calculated by the Lineweaver–Burk plot method.

### 2.6. Preparation of the Protease-Treated MP Samples

MPs were prepared using the method reported by Zhou et al. [19]. The BCA method was employed to adjust the concentration of the MP solution to 5 mg/mL. The protease solution was mixed with MP solution in a 1:1 volume ratio and then incubated at 40 °C for 4 h. Samples of the enzyme-treated MP solution were collected at hydrolysis time points of 0, 0.5, 1, 1.5, 2, 3, and 4 h, and the reaction was terminated by adding an equal volume of 12.5% TCA at the end of the sampling. Treatment of the MP solution with PBS instead of the protease solution served as the control group (CK).

### 2.7. SDS-PAGE 

SDS-PAGE was performed with a 12% separating gel and a 5% stacking gel, as outlined by Zhou et al. [19]. The gel was stained with Coomassie Brilliant Blue R-250 and subsequently destained until the background became clear. The molecular weights of the proteins were estimated by comparing them with colored protein markers ranging from 11 to 245 kDa.

### 2.8. Assessment of Protein Hydrolysis Index (PI)

PI was measured using a fluorometric-based approach as described by Zhou et al. [19]. Fluorescence of enzyme-treated MPs was measured at λex = 375 nm and λem = 475 nm using a 96-well plate spectrofluorometer. PI (%) was expressed as the N-terminal α-amino acid content of the sample as a percentage of the total protein content.

### 2.9. Surface Hydrophobicity

Surface hydrophobicity was assessed following the method described by Li et al. [5]. Treated MP solutions were adjusted to various concentrations (0.2–1.0 mg/mL) by dilution with 0.02 M PBS (pH 7.0). Subsequently, 1 mL samples were combined with 8 mM 1-anilino-8-napthalenesulfonate (ANS) and then incubated at 37 °C for 20 min in a dark environment. The fluorescence intensity of the mixtures was monitored by a microplate reader (Thermo Scientific™ Multiskan SkyHigh) at excitation and emission wavelengths of 365 and 484 nm, respectively. Surface hydrophobicity was expressed as the slope of fluorescence intensity and the concentration of enzyme-treated MP solution.

### 2.10. FTIR and Secondary Structure Determination

The protein structure was analyzed using the methodology of Du et al. [20]. MP solutions after protease treatment for 4 h were freeze-dried. The dried samples (2 mg) were pestled with 100 mg of KBr powder in a mortar, and the tablets were pressed for FTIR spectral scanning after thorough grinding. The scanning area, frequency, and resolution were 400–4000 cm^−1^, 64 times, and 4 cm^−1^, respectively, and KBr at 25 °C was used as the background spectrum. The amide I band (1600–1700 cm^−1^) within the spectral range (400–4000 cm^−1^) was further analyzed by PeakFit 4.12 software. Secondary structure composition was then quantified through multi-peak fitting employing the Lorentz function.

### 2.11. Evaluation of Surface Morphology and Particle Size

Surface morphology characterization was performed using atomic force microscopy (AFM) based on the protocol described by Zhou et al. [21]; 5 μL enzyme-treated MP solution (5 mg/mL) was evenly spread onto a clean-cut mica sheet, air-dried at 25 °C, and subsequently imaged using AFM in light-touch mode. The scanning area (5.0 × 5.0 μm^2^) and the root mean square roughness (Rq) were obtained using NanoScope Analysis 1.7 software.

The particle size of the treated MP solution was determined using a Zetasizer Pro (Malvern Panalytical Ltd, Malvern, UK) at 25 °C. Each group underwent three replicates during particle size analysis.

### 2.12. Free Amino Acids (FAAs) Analysis

Free amino acids (FAAs) analysis was conducted following the protocol reported by Li et al. [5]. Briefly, the supernatant of the samples was filtered through a 0.45 μm filter membrane. The filtrates were analyzed through an automated amino acid analyzer (L-B900, Hitachi, Tokyo, Japan) to measure the amino acid content. The mean of five replicates per group was calculated, and the FAA content in the samples was presented as mg/100 mL. PBS (pH 7.0) buffer instead of the protease solution was defined as the control group.

### 2.13. Metabolite Analysis by LC-MS/MS

The metabolites of enzyme-treated MPs were identified using LC-MS/MS according to the description of Liao et al. [22]. Metabolites of the samples were analyzed using a Thermo Vanquish (Thermo Fisher Scientific, Waltham, MA, USA) UHPLC system associated with a Thermo Orbitrap Exploris 120 MS; 2 μL of the supernatant of MP hydrolysates including 2-Chloro-L-phenylalanine (4 ppm) was injected into an ACQUITY UPLC^®^ HSS T3 (Waters, Milford, MA, USA) column at 40 °C and separated by elution at a constant flow rate of 0.3 mL/min. In the positive ion mode, the mobile phases were 0.1% formic acid–acetonitrile (B2) and 0.1% formic acid aqueous solution (A2), and the gradient elution procedure was as follows: 0–1 min, 8% B2; 1–8 min, 8%–98% B2; 8–10 min, 98% B2; 10–10.1 min, 98%–8% B2; 10.1–12 min, 8% B2. In the negative ion mode, the mobile phases were acetonitrile (B3) and 5 mM ammonium formate (A3), and the gradient elution procedures were 0–1 min, 8% B3; 1–8 min, 8%–98% B3; 8–10 min, 98% B3; 10–10.1 min, 98%–8% B3; 10.1–12 min, 8% B3. The MS was operated in a full-scan mode with positive (ESI+) and negative (ESI−) ionization modes, respectively, with a spray voltage of 3.50 kV for positive ions and −2.50 kV for negative ions. A primary full scan was performed at a resolution of 60,000 with a primary ion scanning range of *m*/*z* 100–1000, and secondary cleavage was performed using HCD with a collision energy of 30% and a secondary resolution of 15,000, with 4 ions fragmented prior to the acquisition of the signal, while dynamic exclusion was used to remove unwanted MS/MS information. Raw data were converted into a common data format file (mzML) and further processed through the MSDIAL software platform (version 4.0), including metabolite number, retention time, *m*/*z*, metabolite name, ionic mode, and peak area. Identification of metabolites via HMDB and METLIN databases was based on the descriptions by Wu et al. [23].

### 2.14. Electronic Tongue Determination

The electronic tongue analysis of MP hydrolysates was performed following the methodology established by Zhou et al. [21]. The supernatant of the sample was analyzed by using the TS-5000Z taste sensor (INSENT, Atsugi, Japan). The results are presented as the average of five repetitions.

### 2.15. Statistical Analyses

All data are shown as mean ± standard deviation. Data on yeast screening indicators, protease activities, enzymatic properties, protein hydrolysis index, surface hydrophobicity, FAA content, and metabolites were analyzed using one-way ANOVA of IBM SPSS Statistics 27. Duncan’s multiple range test was employed to determine significant differences between the means at the 5% significance level. The effect of different concentrations of metal ions and inhibitors on protease activities was evaluated using an independent samples *t*-test at a 5% level of significance for each group. The key metabolites were assessed among these groups by partial least squares discriminant analysis (PLS-DA) using SMICA 14.1.

## 3. Results and Discussion

### 3.1. Biological Identification and Determination of Protease Activities in Yeasts

**Figure 1:**
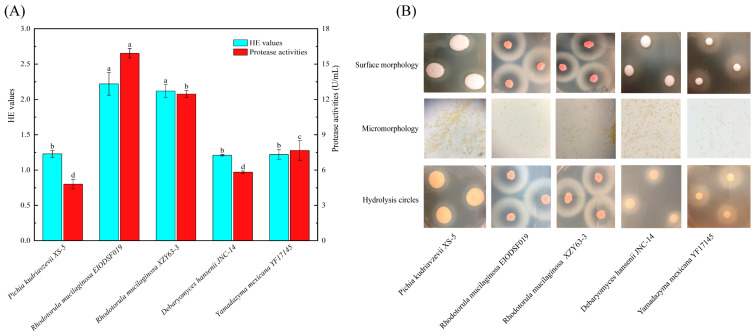
Assessment of the ability of the screened yeasts to hydrolyze proteins (**A**). Morphological characteristics of the screened yeasts (**B**). a–d: Different letters indicate significant differences among groups (*p* < 0.05).

HE (hydrolysis circles diameter/colony diameter) values and protease activities of the yeasts are presented in Figure 1A. *R. mucilaginosa EIODSF019* and *R. mucilaginosa* XZY63-3 exhibited significantly higher HE (2.22 and 2.12, respectively) and protease activities (15.92 and 12.46 U/mL, respectively) compared with *P. kudriavzevii XS-5*, *D. hansenii JNC-14*, and *Y. mexicana YF17145* (*p* < 0.05). The hydrolysis circles, surface morphology, and micromorphology of the yeasts are shown in Figure 1B. All strains displayed an elliptical shape and lacked flagella, indicating that they were classified as yeasts. *R. mucilaginosa EIODSF019* and *R. mucilaginosa* XZY63-3 colonies on YPD agar plates were red and spherical, with pronounced hydrolysis circles, suggesting that they could show dramatic capability in degrading proteins.

A rich and stable enzyme source is the key to improving the taste of dry-cured meat products [24]. Studies have shown that exogenous proteases secreted by microorganisms are better suited to the fermentation conditions in dry-cured meat products compared with endogenous proteases, and that strains with protease activities of more than 10 U/mL can be considered as high enzyme-producing strains [9]. Li et al. [5] showed that *P. aethiopicum* and *P. chrysogenum* showed a high capacity to hydrolyze muscle proteins, and the protease activities of the strains were 12.39 and 11.42 U/mL, respectively. Gong et al. [12] found that *D. hansenii*, *Y. alimentaria*, and *C. zeylanoides* showed significant protease activities to hydrolyze proteins with higher HE values. In this study, the protease activities and HE values of both *R. mucilaginosa* were more than 11 U/mL and 2, respectively, suggesting robust protease secretion conducive to protein hydrolysis during dry-cured ham fermentation. Thus, the extracellular proteases of *R. mucilaginosa EIODSF019* and *R. mucilaginosa XZY63-3* were further characterized.

### 3.2. Effects of pH and Temperature on Yeast Protease Activities

**Figure 2:**
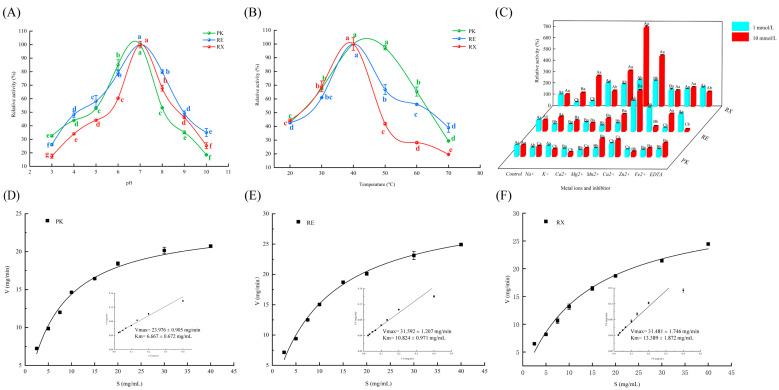
Effects of pH (**A**), temperature (**B**), and metal ions (**C**) on protease activities. The curve and inset represent the non-linear fit of the Michaelis–Menten and Lineweaver–Burk plot of *P. kudriavzevii XS-5* (**D**), *R. mucilaginosa EIODSF019* (**E**), and *R. mucilaginosa XZY63-3* (**F**) proteases. ^A–C^ Different letters indicate significant differences among groups (*p* < 0.05). a–g: Different letters indicate significant differences for different temperatures, pH, and metal ions (*p* < 0.05).

Changes in the relative protease activities of *P. kudriavzevii XS-5* (PK), *R. mucilaginosa EIODSF019* (RE), and *R. mucilaginosa XZY63-3* (RX) at the range of pH (3.0–10.0) and temperature (20–70 °C) are shown in Figure 2A and Figure 2B, respectively. The relative protease activities of PK, RE, and RX all showed a significant increase in the pH from 3.0 to 7.0 and then a decrease in the pH from 7.0 to 10.0. The relative activities of PK, RE, and RX proteases reached a maximum at pH = 7.0. Notably, the proteases of PK and RE maintained relatively high activities (above 50% of the maximum) in the pH range of 5.0–7.0. The relative protease activities of PK, RE, and RX showed a significant increase from 20 °C to 40 °C, followed by a marked decrease from 40 °C to 70 °C. The relative activities of PK, RE, and RX reached the maximum at 40 °C, with no significant difference observed in the maximum relative activities among the three strains. The relative activities of PK, RE, and RX were more than 60% of the maximum at temperatures within 30–40 °C. These findings suggested that the proteases derived from PK, RE, and RX exhibited remarkable adaptability to the change of pH and temperature. This adaptability is a crucial attribute for the practical application of these enzymes. During the processing, dry-cured meat products generally have a low pH value (below 6.0) and a high temperature (30–40 °C), especially for dry-cured hams [5]. The proteases of RE and RX retained over 40% of their maximum activities at pH 5.0. Furthermore, these proteases exhibited relatively high activities within 30–40 °C, offering potential for effective protein degradation during the fermentation process of meat products [25].

### 3.3. Effects of Metal Ions and Inhibitors on Yeast Protease Activities

Metal ions play a pivotal role in regulating the activities of proteases, and they can bind to the active centers of proteases and directly participate in the process of hydrolysis [5]. The effects of metal ions and inhibitors on the relative protease activities of *P. kudriavzevii XS-5* (PK), *R. mucilaginosa EIODSF019* (RE), and *R. mucilaginosa XZY63-3* (RX) are shown in Figure 2C. Compared with PK proteases, the relative protease activities of both RE and RX were promoted when the concentrations of Mg^2+^, Mn^2+^, and Cu^2+^ were 10 mmol/L. It is noteworthy that the RX group exhibited the most significant increase in relative protease activities (*p* < 0.05), which was 212.14%, 595.74%, and 345.65% higher than that of the control group. From the perspective of the mechanism of action, Mg^2+^, Cu^2+^, and Mn^2+^ may help stabilize the protease structure and prevent conformational changes during the catalytic reaction, thereby enhancing the reaction rate [26]. When the concentrations of Ca^2+^ and Zn^2+^ were 10 mmol/L, the relative protease activities of PK and RE exhibited inhibitory effects. The results were also supported by Sun et al. [27], who demonstrated that the activities of extracellular proteases of *Lactobacillus curvatus* isolated from Harbin dry sausages were influenced by Ca^2+^, and a high concentration of Ca^2+^ significantly reduced the protease activities and stabilities. It is noteworthy that the relative protease activities of RE exhibited a promoting effect when the concentration of EDTA was 1 mmol/L. However, at 10 mmol/L, the relative protease activities of RE showed a significant inhibitory effect (*p* < 0.05). This could be attributed to the fact that lower EDTA concentrations can boost protease activities by exposing the hidden active sites. However, EDTA may cause protease denaturation at higher concentrations, leading to a decrease in protease activities [24]. These results indicated that metal ions and EDTA at different concentrations exhibited varying degrees of adaptability to the relative protease activities in PK, RE, and RX, which may be associated with the active sites and properties of the enzymes. Li et al. [5] showed that different concentrations of metal ions and inhibitors exhibited different adaptations to the relative protease activities of *P. aethiopicum* versus *P. chrysogenum*. This also implied that proteases from different strains could exhibit varying sensitivities and response patterns to metal ions, resulting in different hydrolytic capabilities towards proteins.

### 3.4. Analysis of Protease Kinetic Parameters

Figure 2D, Figure 2E and Figure 2F show the Km and Vmax of *R. mucilaginosa EIODSF019* (RE), *R. mucilaginosa XZY63-3* (RX), and *P. kudriavzevii XS-5* (PK) proteases, respectively. The Vmax of PK, RE, and RX proteases were 23.976, 31.592, and 31.481 mg/min, respectively, while the Km values of PK, RE, and RX proteases were 6.667, 10.824, and 13.389 mg/mL, respectively. Compared with RX proteases, lower Km values were observed in RE proteases. Km reflects the affinity of the enzyme for the substrate, with a lower Km value indicating a stronger binding affinity between enzyme and substrate [24]. Vmax reflects the maximum reaction rate, which depends on the enzyme concentration involved in the reaction [24]. The Vmax of RE and RX proteases was significantly higher than that of PK proteases, which implied that the amounts of enzymes needed for hydrolyzing the same proteins were lower in RE and RX groups. Interestingly, the Km of the RE group was lower than that of the RX group, suggesting that RE proteases had a better affinity for the protein substrates. These results indicated that although the proteases of RE and RX could both bind with protein substrates, the protease of RE showed a better affinity capability than that of RX, which could be more helpful for the hydrolysis of MPs.

### 3.5. SDS-PAGE After Protease Treatment of MPs

**Figure 3:**
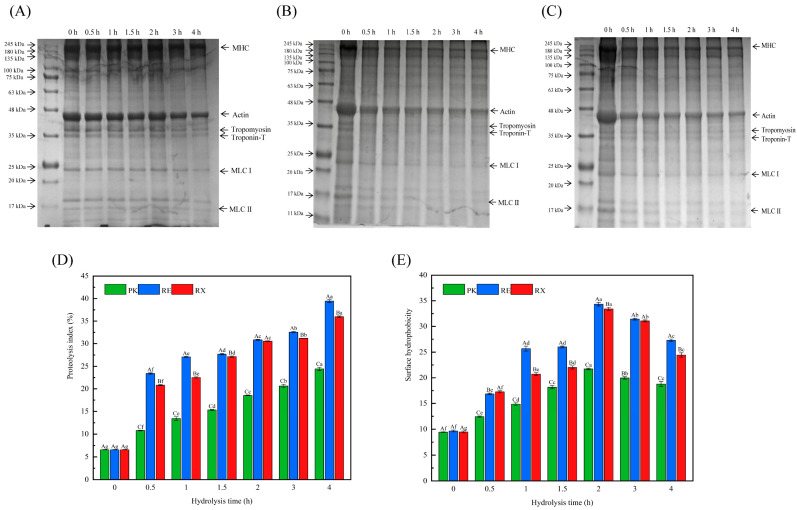
SDS-PAGE changes of myofibrillar proteins treated by proteases of *P. kudriavzevii XS-5* (**A**), *R. mucilaginosa EIODSF019* (**B**), and *R. mucilaginosa XZY63-3* (**C**). The proteolytic index (**D**) and surface hydrophobicity (**E**) of myofibrillar protein treated by proteases. a–g: Different letters indicate significant differences among different hydrolysis times (*p* < 0.05). A–C: Different letters indicate significant differences among groups (*p* < 0.05).

Figure 3A, Figure 3B and Figure 3C show the hydrolysis changes of MPs in gel electrophoresis after a 4 h hydrolysis by the proteases of *P. kudriavzevii XS-5* (PK), *R. mucilaginosa EIODSF019* (RE), and *R. mucilaginosa XZY63-3* (RX), respectively. Compared with the treatment of PK proteases, it was obviously found that the proteases of RE and RX both showed a significant ability to degrade myosin heavy chain (MHC, about 200 kDa), myosin light chain (MLC, 10–30 kDa), tropomyosin (35 kDa), actin (43 kDa), and troponin-T (32 kDa). Interestingly, RE proteases showed more dramatic degradation for actin, MHC, tropomyosin, troponin-T, and MLC. These results suggested that the proteases from different strains exhibited different affinities and hydrolysis capacities for MPs.

The hydrolysis of MPs is the primary factor contributing to the textural changes and taste development in fermented meat products [28]. Actin and myosin are the most abundant proteins in MPs, which are easily degraded into FAAs and small peptides in fermented meat products by protease activities, and are also a key source of taste compounds [9]. Several studies have found that microbial proteases exhibited significant potential for degrading MPs during the processing of dry-cured and fermented meat products [5,29]. Chaves-López et al. [29] showed that proteases secreted by *S. cerevisiae* significantly degraded myosin and actin into Cys, Glu, Lys, and Val. Li et al. [5] showed that microbial proteases could bind to proteins through a specific recognition mechanism and catalyze the hydrolysis reaction of proteins at the binding site. The binding strength between proteases and proteins directly affects the efficiency and rate of the hydrolysis reaction. The present study demonstrated that the RE proteases caused significantly more degradation of myosin and actin than other groups. This observation can be attributed to the fact that with more interaction with myosin and actin, the RE proteases could enhance their degradation to a greater extent.

### 3.6. Analysis of PI Values and Surface Hydrophobicity

Figure 3D and Figure 3E show the evolution of PI values and surface hydrophobicity of MPs after 4 h of protease treatments derived from *P. kudriavzevii XS-5* (PK), *R. mucilaginosa EIODSF019* (RE), and *R. mucilaginosa XZY63-3* (RX), respectively. PI values showed a gradual increase in PK, RE, and RX groups with the increase in incubation time. RE and RX groups exhibited significantly higher PI values than the PK group during the hydrolysis of MPs. Compared with the original PI values (0 h) of MPs, the PI values of MPs in PK, RE, and RX groups increased to 24.42, 39.45, and 35.98% after 4 h of hydrolysis treatment by the proteases, respectively. Among all groups, the highest PI values of MPs were observed in the treatment of RE proteases after 4 h of hydrolysis. The PI values are considered to be a key indicator to evaluate the changes of muscle protein hydrolysis, and higher values in PI indicate that the hydrolysis degree of proteins is significantly higher [5]. Several studies have shown that the degradation of actin, troponin-T, tropomyosin, and myosin was the main contributor to developing the PI values throughout the processing of dry-cured meats [21]. In this study, the treatment of RE exhibited the highest PI values, which could be attributed to the more intense degradation in actin and myosin occurring after the treatment of RE proteases, which was consistent with the SDS-PAGE results.

Surface hydrophobicity is a crucial parameter for assessing changes in protein conformation, aggregation degree, and interactions between proteases and MPs [5]. Surface hydrophobicity of MPs first increased from 0 h to 2 h and then significantly decreased from 2 h to 4 h among all treatments of PK, RE, and RX proteases during the incubation. Among these treatments of PK, RE, and RX proteases, the surface hydrophobicity of MPs showed the highest values in RE throughout the entire hydrolysis process, and the surface hydrophobicity values of MPs in PK, RE, and RX proteases reached 18.78, 27.29, and 24.45 after the hydrolysis of 4 h, respectively. The highest values of surface hydrophobicity of MPs in RE could be explained by the intense hydrolysis of MPs induced by RE proteases, which changed the structure of MPs and exposed their hydrophobic surface. Du et al. [20] showed that the treatment of proteases from *Lactobacillus plantarum* significantly altered the surface hydrophobicity of proteins and decreased the helix structure of proteins. Proteases could recognize and cleave specific peptide bonds of proteins, and then change the original conformation and surface properties. Wang et al. [9] demonstrated that the internal structure of MPs was disrupted under the action of proteases from *Staphylococcus epidermidis*, leading to the exposure of their internal hydrophobic groups, thereby further enhancing the surface hydrophobicity of MPs. Li et al. [5] found that the rearrangement, aggregation, and folding of protein molecules could occur, resulting in the reburial of exposed hydrophobic groups within the molecular interior, which decreased the surface hydrophobicity of MPs treated by *Penicillium* protease. Hence, the reduction in surface hydrophobicity observed in this study during 3 to 4 h in the treatment of PK, RE, and RX proteases could be due to the fact that the hydrolysis of myosin and actin caused the refolding and rearrangement of protein molecules, and then buried the hydrophobic surfaces.

### 3.7. Analysis of FTIR Peak Attribution and Secondary Structure

**Figure 4:**
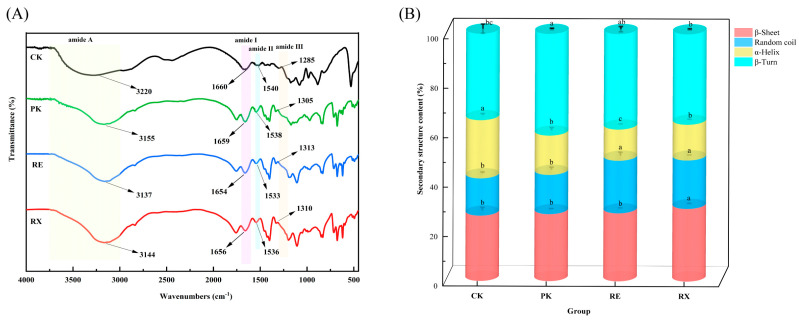
FTIR spectra (**A**) and secondary structure content (**B**) of myofibrillar proteins after treatment with yeast proteases. a–c: Different letters indicate significant differences among groups (*p* < 0.05).

The FTIR spectra of the untreated (CK) and enzyme-treated (PK, RE, and RX) MPs are shown in Figure 4A. Shifts in peak positions were observed in PK, RE, and RX spectra compared with the CK spectra, indicating that protease treatment altered the secondary structure of MPs. Compared with the CK spectra, the characteristic peaks in amide II bands of the PK, RE, and RX spectra shifted to lower wavelengths (from 1540 to 1538, 1533, and 1536 cm^−1^, respectively), which suggested that the enzyme treatment unfolded the MPs after 4 h of hydrolysis treatment [20]. Compared with the spectra of CK, the characteristic peaks of PK, RE, and RX spectra in the amide I band shifted to lower wavelengths (from 1660 to 1659, 1654, and 1656 cm^−1^, respectively). This shift was likely caused by a reduction in the α-helix content of MPs due to enzyme treatment, which weakened hydrogen bonding within the protein molecules and reduced the vibrational energy [20]. Notably, the lowest wavelength of the characteristic peak in the amide I band was observed in the RE spectra, suggesting that RE proteases effectively promoted the hydrolysis of MPs.

The alterations in the secondary structure of MPs are presented in Figure 4B. Compared with CK, the α-helix content in PK, RE, and RX decreased by 8, 11, and 9%, respectively, which weakened the intramolecular hydrogen bonding, and increased the unfolding and flexible and pliable conformation [9]. Notably, the lowest content of α-helix was observed in RE, which was consistent with the result of RE spectra that showed the lowest characteristic peak wavelengths in the amide I band, further suggesting that RE proteases accelerated the hydrolysis of MPs. The content of β-turn was significantly increased in PK compared to CK, which may be due to α-helix mainly converting to β-turn. It is noteworthy that the elevated content of β-turn may conceal the protein cleavage site, which further confirms the change of protein structure that PK exhibited lower PI values in these enzyme-treated groups. Random coil is a characteristic of a disordered structural transition [9]. The highest content of random coil was observed in the RE group among these enzyme-treated groups, indicating that the RE protease may significantly change the structure of MPs. Wang et al. [9] showed that the β-sheet content in MPs treated with *S. epidermidis* proteases decreased significantly, while the proportions of random coils and α-helix increased with the extension of hydrolysis time. Du et al. [20] showed a decrease in α-helical content and an increase in β-sheet content after 3 h of hydrolysis treatment with *L. plantarum* proteases. In this study, the alterations in protein secondary structure content after PK, RE, and RX protease treatments were in agreement with the results of Du et al. [20], but were contrary to the findings of Wang et al. [9]. This may be due to the fact that proteases of different strains showed different specificities and cleavage sites for proteins, resulting in differences in protein secondary structure.

### 3.8. Analysis of Surface Morphology and Particle Size

**Figure 5:**
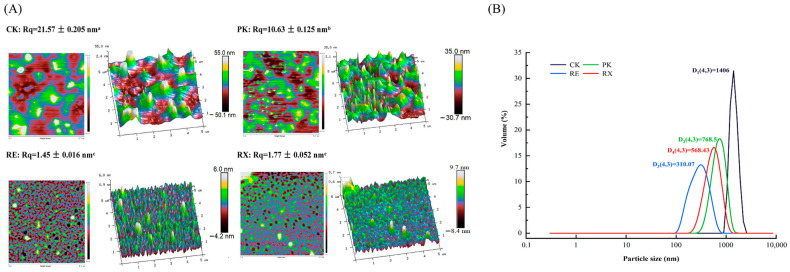
AFM images (**A**) and particle size (**B**) of myofibrillar proteins after treatment with yeast proteases. ^a–c^: Different letters indicate significant differences among groups (*p* < 0.05).

The microstructure of untreated (CK) and enzyme-treated (PK, RE, and RX) MPs is shown in Figure 5A. The untreated (CK) MPs exhibited extensive deposition, presenting a rough surface with numerous protrusions, as observed in both 2D and 3D images. Compared with CK, the protrusions of MPs in PK, RE, and RX were obviously reduced, and the heights of MPs in PK, RE, and RX significantly decreased (*p* < 0.05). Additionally, the Rq values, which represent a parameter for characterizing surface roughness, were calculated based on the height distribution of all points across the entire region [9]. The Rq values of CK, PK, RE, and RX were 21.57, 10.63, 1.45, and 1.77 nm, respectively. Notably, RE exhibited the lowest Rq values, suggesting that RE proteases were more effective at hydrolyzing MPs into small-molecule proteins or other small-molecule substances [9].

Wang et al. [24] showed that peptides and amino acids were produced during hydrolysis of meat proteins, which could develop new structures or aggregates on the protein surface. These new structures or aggregates may present different morphological features, such as granule, agglomerate, or reticulate in AFM images, and change the protein particle size [9]. To further investigate the changes in MP particle size among enzyme-treated groups, Zetasizer Pro was used to analyze the particle size of MPs. D (4, 3) represents the volume-averaged particle size of the proteins. As illustrated in Figure 5B, compared with CK, the D (4, 3) values from 1486.00 nm of original MP significantly decreased to 768.50, 310.04, and 568.43 nm in PK, RE, and RX, respectively. Wang et al. [24] showed that the presence of *S. epidermidis* proteases was able to disrupt the chemical bonds between MPs, and weaken the aggregation structure, resulting in a significant decrease in MP particle size, which inhibited protein reaggregation. Notably, the lowest D (4, 3) values were observed in RE, further suggesting that RE proteases significantly inhibited the reaggregation of MPs, allowing intact MP structures to be degraded into homogeneous small molecules. The changes in particle size of MPs further implied that RE proteases showed great potential to hydrolyze meat proteins.

### 3.9. FAA Analysis

**Figure 6:**
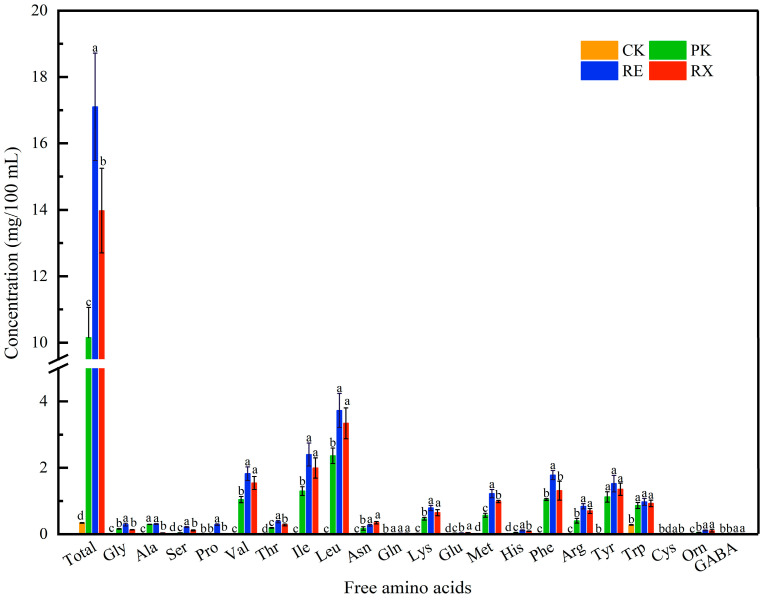
Changes in free amino acid contents of myofibrillar protein hydrolyzed by *P. kudriavzevii XS-5*, *R. mucilaginosa EIODSF019*, and *R. mucilaginosa XZY63-3* proteases. a–d: Different letters indicate significant differences among groups (*p* < 0.05).

Changes of FAA content in untreated (CK) and enzyme-treated (PK, RE, and RX) MPs are shown in Figure 6. The total FAA content significantly (*p* < 0.05) increased from 0.34 mg/100 mL in CK to 10.15, 17.10, and 13.98 mg/100 mL in PK, RE, and RX, respectively. The total FAA content of RE and RX was 1.68 and 1.38-fold higher than that of PK, respectively. The content (*p* < 0.05) of Val, Ile, Leu, Met, Phe, Tyr, and Trp significantly increased in enzyme-treated groups. Val, Ile, and Leu showed highly relative profiles in PK, RE, and RX groups, respectively, and the content of these three amino acids accounted for more than 46% of the total content in each enzyme-treated group. This implied that enzyme-treated MPs markedly promoted the production of Val, Ile, and Leu, further indicating that these enzymes may possess selectivity and higher catalytic efficiency towards specific amino acids. Zhou et al. [21] showed that the protease activities of different strains varied, which directly affected the rate and efficiency of MP degradation. Li et al. [5] found that the more intense binding of *P. aethiopicum* proteases to MPs could accelerate the generation of FAAs in comparison with *P. chrysogenum* proteases. In these enzyme-treated groups, the highest levels of total FAAs were observed in RE, suggesting that RE showed a stronger binding capacity to MPs, accelerating the hydrolysis of MPs and promoting the production of FAAs. Wang et al. [9] showed that *S. epidermidis* proteases accelerated the hydrolysis of MPs and notably increased the production of Glu and Ala. It was noteworthy that RE proteases showed a significant effect on the accumulation of Val, Ile, and Leu in the present study, which differed from the results of Wang et al. [9]. The variations in FAA content could be due to the fact that proteases from different strains may have specific cleavage sites in proteins and then accelerate the release of specific amino acids.

### 3.10. Metabolite Analysis

**Figure 7:**
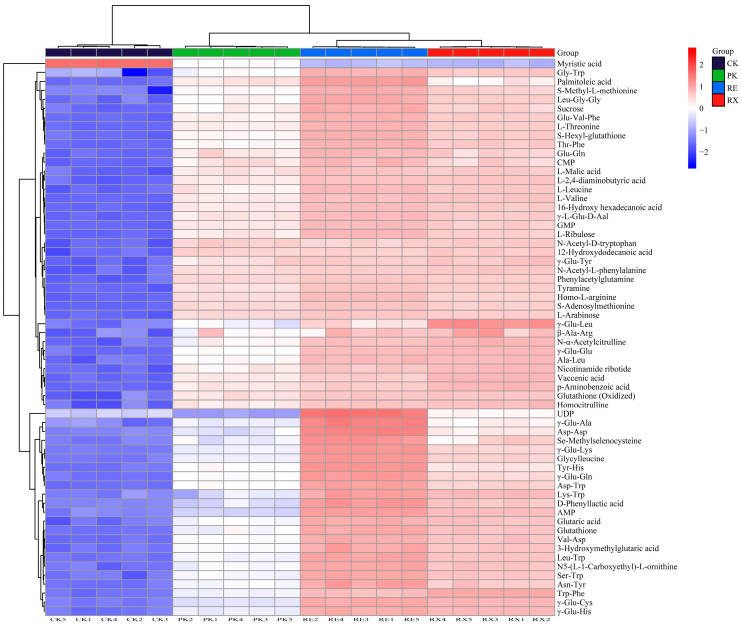
The cluster heat map of metabolites derived from myofibrillar proteins after treatment with yeast proteases.

To characterize the changes of metabolites in untreated (CK) and enzyme-treated (PK, RE, and RX) MPs, the metabolites analyzed by LC-MS/MS are presented in Table S1. A total of 62 metabolites were identified, including peptides (28), amino acid derivatives (14), organic acids (11), nucleotides (6), and sugars (3). Compared with the untreated group, the metabolite content significantly increased among these enzyme-treated groups (*p* < 0.05). It was noteworthy that the highest levels of metabolites were observed in the RE group among all enzyme-treated groups. The total content of peptides, amino acid derivatives, organic acids, nucleotides, and sugars in the RE group increased by 3.26, 1.31, 1.16, 3.30, and 1.07 times, respectively, compared with the PK group. To further characterize the differences in the content of all metabolites among these groups, a clustered heatmap analysis is presented in Figure 7. CK, PK, RE, and RX groups showed disparate color distributions, indicating distinctive differences in metabolites occurring among these groups. The CK group was clustered into a separate category, differing from the RE, RX, and PK groups. Interestingly, although the enzyme-treated MPs (PK, RE, and RX) clustered into one category, the different enzyme-treated groups further clustered into corresponding subcategories. These results suggested that different strain proteases could be the key factor causing differences in metabolite levels of MPs [21].

To further evaluate the difference of metabolites among these groups, PLS-DA analysis was carried out, and the values of R2X (cum) and Q2 (cum) in PLS-DA were 0.991 and 0.995, respectively. The Biplot loading plot (Figure S2A) showed that significant differences were observed between untreated and enzyme-treated groups, and that a large number of metabolites were close to the RE group. Variable Importance for the Projection (VIP) in the PLS-DA model helps identify metabolites, and the metabolites with VIP more than 1 are considered as the key source of the difference among these groups. As illustrated in Figure S2B, the VIP values of Trp-Phe, Myristic acid, N-Acetyl-D-tryptophan, 12-Hydroxydodecanoic acid, γ-Glu-Leu, Palmitoleic acid, γ-Glu-Glu, UDP, p-Aminobenzoic acid, γ-Glu-Lys, Vaccenic acid, γ-Glu-Tyr, Tyr-His, N-α-Acetylcitrulline, γ-Glu-His, γ-Glu-Cys, Nicotinamide ribotide, γ-Glu-Ala, γ-Glu-Gln, N-Acetyl-L-phenylalanine, and Lys-Trp were more than 1, suggesting that these metabolites could play a crucial role in distinguishing the metabolite differences among these groups.

Amino acid derivatives and peptides primarily originate from protein hydrolysis and are closely associated with the specificity of microbial proteases [21]. In the present study, amino acid derivatives (N-Acetyl-D-tryptophan, N-α-Acetylcitrulline, and N-Acetyl-L-phenylalanine) and peptides (Trp-Phe, Tyr-His, Lys-Trp, γ-Glu-Leu, γ-Glu-Glu, γ-Glu-Lys, γ-Glu-Tyr, γ-Glu-His, γ-Glu-Cys, γ-Glu-Ala, and γ-Glu-Gln) were significantly different among these groups (*p* < 0.05). The total content of amino acid derivatives in RE and RX was 1.34-fold and 1.53-fold of that of PK, respectively. Notably, peptides were the most abundant of the metabolites and accounted for more than 52% of the differential metabolites. The content of peptides in RE and RX was 5.94-fold and 3.77-fold compared with PK, respectively, and γ-glutamyl peptides accounted for more than 72% of these peptides. Among the metabolites, the total content of γ-glutamyl peptides was 64.51, 174.52, 1036.24, and 657.91 ng/mL in the CK, PK, RE, and RX, respectively. These results suggested that peptides and amino acid derivatives were accumulated significantly in RE. Different proteases show different catalytic properties and substrate selectivity, which determine the efficiency and selectivity of peptide bond cleavage in MPs [5]. Wu et al. [23] showed that *Staphylococcus vitulinus* and *Staphylococcus xylosus* proteases were able to hydrolyze proteins in dry-cured bacon, accelerating the release of γ-glutamyl peptides (γ-Glu-Glu and γ-Glu-Cys) and FAAs. In this study, the highest levels of γ-glutamyl peptides were observed in RE, further suggesting that differences in metabolite components and levels were closely related to the differences in strain proteases.

Liao et al. [22] showed that organic acids and nucleotides responded positively to sourness and umami to some extent. Furthermore, organic acids and nucleotides showed relatively higher taste-active thresholds compared with most FAAs and peptides. In this study, the total content of myristic acid, 12-hydroxydodecanoic acid, palmitoleic acid, and vaccenic acid in RE and RX groups was 1.39-fold and 1.59-fold higher than that of PK, respectively. The total content of UDP and nicotinamide ribotide in RE and RX groups was 2.86-fold and 1.69-fold higher than that of PK, respectively. Although the levels of nucleotides and organic acids were higher in RE and RX groups than in the PK group, the increase of nucleotides and organic acids in RE and RX was markedly lower than that of peptides (*p* < 0.05). These findings implied that nucleotides and organic acids could contribute to the taste development of enzyme-treated MPs to a minor extent compared with peptides.

### 3.11. The Changes of Taste Attributes of Enzyme-Treated MPs

**Figure 8:**
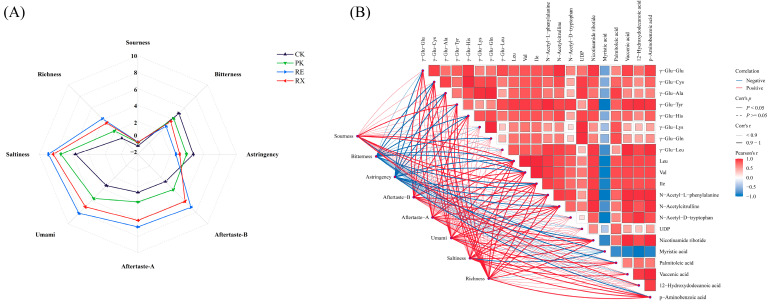
Sensory scores of myofibrillar proteins after treatment with yeast proteases (**A**) and the correlation between key metabolites and taste attributes (**B**).

Taste changes in untreated (CK) and enzyme-treated (PK, RE, and RX) MPs are shown in Figure 8A. The intensity of saltiness, umami, richness, Aftertaste-A, and Aftertaste-B significantly increased, while the intensity of bitterness and astringency significantly decreased in PK, RE, and RX compared with the CK. Compared with the CK group, the intensities of bitterness and astringency both reduced by more than 17%, 43%, and 27% in PK, RE, and RX, while aftertaste intensities increased by 0.43, 1.58, and 1.25-fold, and richness intensities increased by 1.64, 4.36, and 3.40 times, respectively. The electronic tongue results further revealed that MPs treated with proteases from RE showed the most favorable taste profiles compared with other groups.

Aftertaste, saltiness, umami, richness, and bitterness are key characteristics that are commonly used to evaluate the quality of meat products [21]. FAAs and amino acid derivatives can collectively contribute to the unique taste of meat products by participating in chemical reactions and interacting with other taste compounds [30]. Val, Ile, and Leu may bring out bitterness and sourness, and decrease the desirable attribute of the overall taste. N-Acetyl-D-tryptophan, N-α-Acetylcitrulline, and N-Acetyl-L-phenylalanine could be associated with umami [30]. Nucleotides and organic acids could show umami and sourness at low concentrations [22]. Taste-active peptides serve as the primary contributors to taste in many fermented meat products and impart kokumi, bitterness, or umami sensation, and they have a significant impact on modulating taste attributes. γ-Glutamyl peptides contribute significantly to taste attributes because of their abilities to adjust the levels of umami, richness, sweetness, and saltiness [31]. Aftertaste and richness are important indicators for assessing the intensity of the kokumi [32]. Li et al. [33] showed that the changes of γ-glutamyl peptides improved the taste attributes of protein hydrolysis products. Wang et al. [32] showed that γ-glutamyl peptides such as γ-Glu-Phe, γ-Glu-Leu, γ-Glu-Val, and γ-Glu-His inhibited bitterness and triggered kokumi sensation. Xia et al. [34] demonstrated that γ-glutamyl peptides significantly boosted the perception of saltiness and umami. In this study, γ-Glu-Leu, γ-Glu-Glu, γ-Glu-Lys, γ-Glu-Tyr, γ-Glu-His, γ-Glu-Cys, γ-Glu-Ala, and γ-Glu-Gln were the key differential metabolites in terms of peptides, and the highest content of these γ-glutamyl peptides was detected in the RE group compared with the PK and RX groups. These results further confirmed the highest intensity of aftertaste and richness observed in RE.

### 3.12. Correlation Between Key Metabolites and Taste Properties

Pearson correlation analysis further explored the relationship among key metabolites, amino acids, and taste characteristics. As illustrated in Figure 8B, several metabolites showed highly positive correlations with the sensory parameters of umami, richness, and aftertaste. Specifically, Val (r = 0.97, *p* < 0.05), Ile (r = 0.96, *p* < 0.05), Leu (r = 0.95, *p* < 0.05), N-Acetyl-L-phenylalanine (r = 0.93, *p* < 0.05), γ-Glu-Tyr (r = 0.93, *p* < 0.05), γ-Glu-Cys (r = 0.92, *p* < 0.05), and Nicotinamide ribotide (r = 0.93, *p* < 0.05) were highly and positively correlated with umami. γ-Glu-Cys (r = 0.97, *p* < 0.05), γ-Glu-His (r = 0.96, *p* < 0.05), γ-Glu-Glu (r = 0.93, *p* < 0.05), γ-Glu-Ala (r = 0.91, *p* < 0.05), and Ile (r = 0.93, *p* < 0.05) were highly and positively correlated with aftertaste. γ-Glu-Cys (r = 0.94, *p* < 0.05), γ-Glu-His (r = 0.94, *p* < 0.05), γ-Glu-Glu (r = 0.92, *p* < 0.05), γ-Glu-Ala (r = 0.91, *p* < 0.05), and N-α-Acetylcitrulline (r = 0.94, *p* < 0.05) were highly and positively correlated with richness. These findings further verified that FAAs and amino acid derivatives had the potential to enhance umami, and that γ-glutamyl peptides were strongly associated with richness and aftertaste, aligning with the results reported by Li et al. [33]. Notably, a large number of γ-glutamyl peptides were found in metabolites with the highest positive correlations with umami, richness, and aftertaste, further suggesting that γ-glutamyl peptides play a pivotal role in enhancing the taste of protein hydrolysates. Organic acids and nucleotides were not strongly correlated with umami, richness, and aftertaste, further suggesting that organic acids and nucleotides may not be the main metabolites in distinguishing taste differences of protein hydrolysates among these groups.

## 4. Conclusions

This work clearly demonstrated that *R. mucilaginosa EIODSF019* (RE) and *R. mucilaginosa XZY63-3* (RX) proteases were intensely responsive to the degradation of MPs. Compared with *P. kudriavzevii XS-5* (PK) and RX proteases, RE proteases exhibited higher hydrolysis capacity for myosin and actin, which could be due to a lower Km value and a higher Vmax value of RE proteases. The extensive hydrolysis of MPs promoted the accumulation of FAAs and metabolites. LC-MS/MS demonstrated that γ-glutamyl peptides were the most abundant components of MP hydrolysates by *R. mucilaginosa EIODSF019*. PLS-DA and correlation network further revealed that the accumulation of γ-Glu-Leu, γ-Glu-Glu, γ-Glu-Lys, γ-Glu-Tyr, γ-Glu-His, γ-Glu-Cys, γ-Glu-Ala, and γ-Glu-Gln were mainly responsible for the enhancement of aftertaste, richness, and umami after the treatment of RE.

## Data Availability

Data supporting the findings of this study are available from the corresponding author upon reasonable request.

## Supplementary Materials

The following supporting information can be downloaded at https://www.mdpi.com/article/10.3390/foods14111867/s1, Figure S1: Phylogenetic tree of *Rhodotorula mucilaginosa EIODSF019* (A), *Rhodotorula mucilaginosa XZY63-3* (B), *Debaryomyces hansenii JNC-14* (C), and *Yamadazyma mexicana YF17145* (D); Figure S2: Biplot loadings (A) and Variable Importance for the Projection (B) of metabolites of myofibrillar proteins after 4 h of treatment with proteases from *P. kudriavzevii XS-5*, *R. mucilaginosa EIODSF019* and *R. mucilaginosa XZY63-3*; Table S1: Metabolite content (ng/mL) in untreated and protease-treated MPs.

## Abbreviations

The following abbreviations are used in this manuscript:

MP	Myofibrillar protein
RE	*Rhodotorula mucilaginosa EIODSF019*
RX	*Rhodotorula mucilaginosa XZY63-3*
PK	*Pichia kudriavzevii XS-5*
TCA	Trichloroacetic acid
PI	Protein hydrolysis index
FTIR	Fourier Transform Infrared Spectroscopy
FAA	Free amino acids
SDS-PAGE	Sodium Dodecyl Sulfate-Polyacrylamide Gel Electrophoresis
