# Supplementary material for: LC-MS/MS-Based Metabolomics and Multivariate Statistical Analysis Reveal the Mechanism of Rhodotorula mucilaginosa Proteases on Myofibrillar Protein Degradation and the Evolution of Taste Compounds
Source: Foods. 2025 May 24;14(11):1867. doi: 10.3390/foods14111867 (PMC12154007; doi:10.3390/foods14111867)
Supplement: Supplementary file 1 [file foods-14-01867-s001.zip › foods-3622885-supplementary.pdf]

Supplementary materials:

Figure S1. Phylogenetic tree of *Rhodotorula mucilaginosa EIODSF019* (**A**), *Rhodotorula mucilaginosa XZY63-3* (**B**), *Debaryomyces hansenii JNC-14* (**C**) and *Yamadazyma mexicana YF17145* (**D**).

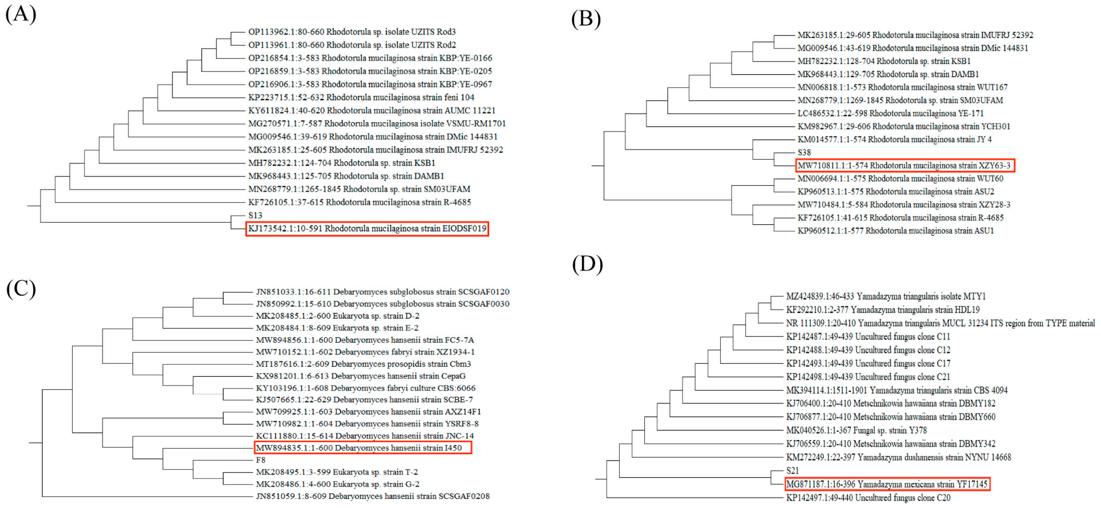

Figure S2. Biplot loadings (**A**) and Variable Importance for the Projection (**B**) of metabolites of myofibrillar proteins after 4 h of treatment with proteases from *P. kudriavzevii XS-5*, *R. mucilaginosa EIODSF019* and *R. mucilaginosa XZY63-3*.

(A) (B)

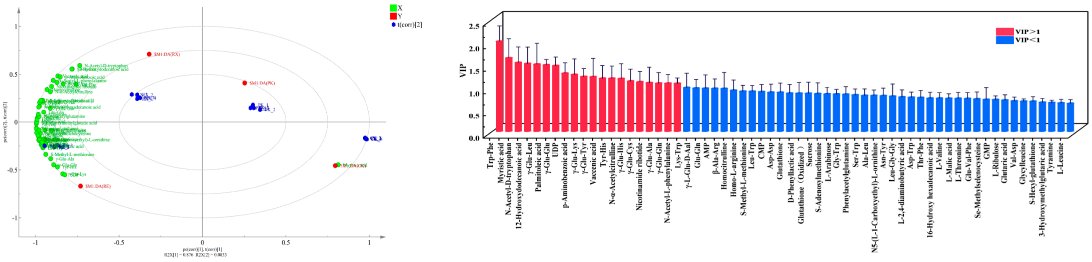

Table S1. Metabolite content (ng/mL) in untreated and protease-treated MP.

Metabolites	Groups			
	CK	PK	RE	RX
γ-Glu-Glu	0.05±0.02^{d}	8.10±0.39^{c}	66.74±2.15^{b}	74.03±1.07^{a}
γ-Glu-Cys	0.82±0.15^{c}	12.67±0.83^{c}	364.22±17.92^{a}	244.57±6.73^{b}
γ-Glu-Ala	2.79±0.46^{d}	5.68±0.22^{c}	15.89±0.62^{a}	7.30±0.41^{b}
γ-Glu-Tyr	3.26±0.32^{c}	17.90±2.04^{b}	21.34±0.56^{a}	22.16±1.19^{a}
γ-Glu-His	0.03±0.01^{c}	2.02±0.31^{c}	94.12±11.12^{a}	63.36±2.20^{a}
γ-Glu-Lys	0.04±0.004^{d}	0.74±0.07^{c}	16.42±0.60^{a}	3.37±0.18^{b}
γ-Glu-Gln	3.31±0.37^{d}	45.18±2.06^{c}	358.51±6.67^{a}	113.50±3.84^{b}
γ-Glu-Leu	54.21±2.93^{d}	82.23±4.19^{c}	99.00±4.16^{b}	129.62±2.69^{a}
γ-L-Glu-D-Aal	14.53±0.72^{d}	88.80±4.87^{c}	134.42±3.86^{a}	115.31±2.84^{b}
Glutathione	0.07±0.03^{d}	1.28±0.34^{c}	9.82±0.30^{a}	5.67±0.36^{b}
Glutathione (Oxidized)	0.01±0.011^{c}	3.23±0.63^{b}	7.53±0.43^{a}	7.60±0.38^{a}
S-Hexyl-glutathione	2.86±0.312^{d}	25.50±0.55^{c}	68.13±1.24^{a}	45.76±2.06^{b}
Thr-Phe	0.35±0.04^{d}	15.37±0.93^{c}	76.14±2.62^{a}	45.50±2.88^{b}
Gly-Trp	0.04±0.02^{d}	0.29±0.05^{c}	1.75±0.11^{a}	1.11±0.05^{b}
Ser-Trp	0.48±0.112^{d}	4.34±0.20^{c}	25.38±0.95^{a}	15.94±0.60^{b}
Tyr-His	0.08±0.007^{d}	0.84±0.09^{c}	5.03±0.19^{a}	1.36±0.03^{b}
Glu-Gln	0.14±0.01^{d}	1.06±0.16^{c}	1.76±0.10^{a}	1.33±0.15^{b}
Lys-Trp	0.02±0.008^{c}	0.15±0.06^{c}	2.32±0.37^{a}	1.40±0.06^{b}
Trp-Phe	0.01±0.001^{d}	0.16±0.01^{c}	1.26±0.04^{b}	2.27±0.02^{a}
Val-Asp	0.26±0.02^{d}	1.45±0.06^{c}	4.34±0.08^{a}	3.32±0.11^{b}
Asp-Asp	1.06±0.02^{d}	1.53±0.09^{c}	2.77±0.03^{a}	1.87±0.04^{b}
Ala-Leu	8.78±1.11^{c}	34.82±3.12^{b}	66.07±2.06^{a}	65.10±1.26^{a}
Leu-Trp	1.32±0.12^{d}	17.61±1.07^{c}	157.97±1.98^{a}	84.97±2.20^{b}
Asn-Tyr	0.17±0.02^{d}	1.39±0.16^{c}	7.36±1.05^{a}	3.93±0.52^{b}
Asp-Trp	2.15±0.05^{d}	9.50±0.34^{c}	24.78±0.72^{a}	14.91±0.54^{b}
Leu-Gly-Gly	0.42±0.15^{d}	6.86±1.20^{c}	31.20±1.78^{a}	16.59±0.42^{b}
Glu-Val-Phe	0.07±0.008^{d}	3.13±0.24^{c}	13.13±0.73^{a}	7.40±0.32^{b}
β-Ala-Arg	1.11±0.23^{c}	3.47±0.87^{b}	4.85±0.75^{a}	5.58±0.91^{a}
Total	98.45±3.09^{d}	395.32±7.36^{c}	1682.26±16.95^{a}	1104.83±12.95^{b}
L-Threonine	8.21±0.64^{d}	376.41±11.96^{c}	1360.75±9.05^{a}	756.81±6.59^{b}
L-Valine	45.89±1.67^{d}	746.26±34.46^{c}	1448.20±26.50^{a}	1328.26±10.48^{b}
L-Leucine	20.40±3.01^{d}	377.98±63.99^{c}	902.81±42.15^{a}	686.21±11.76^{b}
Tyramine	0.007±0.002^{d}	9.23±0.73^{c}	29.67±0.63^{a}	19.89±0.51^{b}
Glycylleucine	178.15±5.43^{d}	357.89±3.87^{c}	743.94±6.39^{a}	528.56±8.04^{b}
Homo-L-arginine	0.04±0.007^{d}	24.24±1.03^{c}	67.51±1.35^{a}	35.10±1.85^{b}
Homocitrulline	0.35±0.14^{d}	5.02±0.49^{c}	8.75±0.20^{b}	9.59±0.71^{a}
Phenylacetylglutamine	0.02±0.01^{d}	67.51±3.61^{c}	135.52±3.55^{a}	97.86±1.47^{b}
S-Methyl-L-methionine	0.37±0.16^{d}	10.91±0.51^{c}	66.69±2.50^{a}	28.99±2.33^{b}
Se-Methylselenocysteine	46.43±1.33^{d}	74.00±4.92^{c}	126.27±2.60^{a}	94.11±9.31^{b}
N5-(L-1-Carboxyethyl)-L-ornithine	0.37±0.07^{d}	3.30±0.28^{c}	18.61±0.53^{a}	11.52±0.57^{b}
N-Acetyl-L-phenylalanine	2.92±0.88^{d}	138.31±3.22^{c}	188.43±2.29^{b}	215.09±2.73^{a}
N-α-Acetylcitrulline	0.46±0.12^{d}	5.72±1.16^{c}	13.93±0.56^{b}	16.71±1.15^{a}

Table S1. *Cont.*

Metabolites	Groups			
	CK	PK	RE	RX
N-Acetyl-D-tryptophan	0.07±0.02[c]	30.14±4.34[ab]	27.74±3.73[b]	35.40±3.01[a]
Total	303.68±6.87[d]	2226.93±85.6[c]	5138.81±68.83[a]	3864.10±25.75[b]
Myristic acid	108.56±5.48[a]	4.86±0.58[b]	1.21±0.13[b]	1.02±0.18[b]
Palmitoleic acid	29.10±0.75[c]	71.71±2.16[b]	104.03±1.87[a]	67.02±4.92[b]
Vaccenic acid	13.71±1.66[d]	176.53±4.21[c]	267.56±8.95[b]	355.90±6.08[a]
Glutaric acid	12.44±2.14[d]	55.36±4.17[c]	155.83±4.43[a]	123.01±4.75[b]
L-Malic acid	6.82±1.91[d]	249.02±24.87[c]	475.82±37.59[a]	407.02±14.67[b]
L-2,4-diaminobutyric acid	5.26±0.97[d]	95.43±6.52[c]	179.08±7.05[a]	168.01±6.62[b]
3-Hydroxymethylglutaric acid	228.20±20.28[d]	827.89±7.17[c]	2139.79±183.84[a]	1653.01±31.40[b]
12-Hydroxydodecanoic acid	0.35±0.11[c]	47.67±3.41[b]	46.75±2.12[b]	56.09±2.84[a]
16-Hydroxy hexadecanoic acid	14.41±0.97[d]	239.15±7.45[c]	451.64±7.88[a]	383.52±14.46[b]
p-Aminobenzoic acid	1.19±0.11[d]	41.44±4.10[c]	69.94±3.73[b]	98.18±6.06[a]
D-Phenyllactic acid	1.70±0.04[d]	4.49±0.89[c]	24.53±1.52[a]	14.24±0.69[b]
Total	421.74±19.79[d]	1813.54±32.75[c]	3916.18±204.11[a]	3327.02±51.74[b]
CMP	0.43±0.04[c]	12.64±2.44[b]	28.67±3.82[a]	16.99±3.67[b]
AMP	27.92±3.55[d]	85.88±2.78[c]	752.06±8.44[a]	472.67±3.41[b]
UDP	74.13±2.42[d]	59.32±1.64[c]	173.37±3.41[a]	97.61±1.53[b]
GMP	4.06±0.32[d]	104.91±2.51[c]	231.43±5.16[a]	166.49±10.37[b]
S-Adenosylmethionine	0.01±0.001[d]	24.26±1.99[c]	58.28±3.10[a]	35.03±1.77[b]
Nicotinamide ribotide	0.22±0.04[d]	3.85±0.62[c]	7.36±0.37[b]	8.94±0.56[a]
Total	106.77±3.95[d]	290.86±3.31[c]	1251.16±7.42[a]	797.72±14.10[b]
Sucrose	3.32±0.28[d]	11.29±0.95[c]	19.01±0.66[a]	14.57±0.78[b]
L-Arabinose	0.23±0.046[d]	243.07±7.10[c]	470.04±12.64[a]	348.35±7.28[b]
L-Ribulose	0.49±0.05[d]	33.38±4.37[c]	105.51±2.92[a]	62.72±2.45[b]
Total	4.03±0.0003[d]	287.74±9.46[c]	594.55±13.47[a]	425.63±4.56[b]

Note: Different letters [a-d] indicate significant differences in different sample groups, respectively ($p<0.05$).
